# Clinical Evaluation of Coronally Advanced Flap With or Without Advance-Platelet Rich Fibrin Membrane in the Treatment of Miller’s Class-II Localized Gingival Recession: A Clinical Study

**DOI:** 10.7759/cureus.34919

**Published:** 2023-02-13

**Authors:** Anuja Jagtap, Sachin B Mangalekar, Pallavi Kamble

**Affiliations:** 1 Department of Periodontology, Bharati Vidyapeeth Dental College and Hospital, Sangli, IND

**Keywords:** periodontal treatment, gingival thickness, localized gingival recession, advance-platelet rich fibrin, coronally advanced flap

## Abstract

Introduction: Periodontal treatment focuses on maintaining a patient's natural teeth and gums. The gingival margin recedes to a point apical to the tooth in 20%-100% of people. Coronally advanced flap (CAF) is one of several effective treatments for this condition. This surgery covers the tooth root with gingiva. The predictability of this surgery depends on the blood supply, donor tissue, and surgical skills. Platelet concentrates, which include platelet-rich fibrin (PRF), majority of the time is used for various regenerative therapies. Since no bovine thrombin or anticoagulant is needed, its manufacturing is simpler, cheaper, and less biochemically modified than PRP. Platelet-rich fibrin (PRF) is a fibrin matrix that progressively releases platelet cells and cytokines.

Aim: The present study aimed to evaluate the efficacy of CAF with and without A-PRF in the treatment of Miller’s class-II localized gingival recession.

Materials and Methods: Twenty patients were chosen who had Miller's class-II localized gingival recession. A random number generator was used to place patients into either the "test" or "control" group. Treatment for both Groups A and B included a coronally advanced flap, but only Group A additionally got autologous platelet-rich fibrin (A-PRF). After receiving a detailed explanation of the treatment process, the patient signed an informed consent form. Complete medical and dental histories were taken to see whether there were any absolute or relative contraindications.

Results: Following treatment with either method in the current study, gingival thickness improved considerably. The percentage of root coverage did not change considerably between the two groups. The clinical result might likely have been different if other factors, such as platelet concentration and PRF consistency, had been examined in the current investigation. Furthermore, there was no histological examination of the healing process. As a result, we are unsure of the extent to which PRF affects how effectively connective tissue attaches.

Conclusion: The additional use of A-PRF membrane did not provide additional benefits in terms of root coverage outcomes compared with CAF alone. The use of A-PRF membranes significantly reduced the recession depth.

## Introduction

Periodontal therapy improves periodontal health, extending tooth life. Modern dental care emphasizes aesthetics, and there are many techniques to maintain or improve a patient's smile. [1]. Anterior region gingival recessions are typically treated using periodontal plastic surgery (PPS) [2]. 20%-100% of people have gingival recession, which is an apical migration of marginal soft tissue for tooth or dental implant platform [3,4]. A gingival recession occurs when the gingival border recedes apically from the cementoenamel junction, and the root is exposed [5].

Toothbrushing with a high pressure, by use of hard bristles and longer duration of brushing causes gingiva to recede surrounding the teeth [3]. Root-coverage surgeries relieve dentinal hypersensitivity and eliminate recession-related cosmetic issues [6]. Root coverage may be done for aesthetics, to relieve sensitivity, to prevent or cure root caries and cervical abrasion, to improve restorative outcomes, or to manage plaque [7]. Recent root-covering surgical investigations show success if biological conditions are satisfied [8]. Since the mid-1900s, several ways have been developed to cover exposed roots. Different methods have been recommended to cover denuded root surfaces, including free autogenous grafts, pedicle grafts with rotational flaps, coronal advanced flaps (CAFs), and semilunar flaps. Later studies suggested treating mucogingival deficiencies with Guided tissue regeneration (GTR) membranes and autologous or allogeneic grafts [9].

Coronally advanced flap (CAF) surgery is based on the coronal movement of soft tissue on the exposed root surface. The technique's perfect root coverage, color mixing, and soft tissue margin morphology restoration prove its efficacy and predictability [10]. CAF requires no graft harvesting, making it simpler and less invasive. Long-term CAF-only root coverage is unstable. Root coverage declined from 89.0% at one-month post-op to 58.8% after six months [11]. Therefore, CAF is commonly blended with other regeneration materials or biological components. This approach reconstructs the junctional epithelium and connective tissue, although histologically, the bone repair is minimal. CAF-induced connective tissue attachment is temporary; hence several methods have been used to expedite healing and enhance clinical outcomes [12]. Tissue design and regenerative medicine employ platelet-rich fibrin, plasma, human fibroblast-determined dermal substitute, acellular dermal grids, connective tissue grafts, and many other materials [13].

Autologous platelet concentrates (APCs) have become a potential regenerative substance that may be used alone or in conjunction with other treatments. Soft tissue repair requires APC, according to research [13]. For cytokines like transforming growth factor-1 (TGF-1), vascular endothelial growth factor (VEGF), insulin-like growth factor (IGF), platelet-derived growth factor-stomach muscle (PDGF-sm), and interleukin-1 (IL-1) are available through this platelet concentrate [14]. Platelet-rich plasma (PRP), platelet-rich fibrin (PRF), and concentrated growth factor (CGF) are the three primary generations of APCs. Anticoagulants, thrombin, and calcium chloride promote fibrin clump at surgical sites. Second-generation platelet concentration includes PRF. Its manufacture is simpler and cheaper than PRP since no bovine thrombin or anticoagulant is needed. Platelet-rich fibrin slowly releases cytokines (growth factors) and cells (PRF). This membrane is useful as an intercellular barrier and resorbable interposition membrane. The PRF layer inhibits gingival epithelium invagination by blocking epithelial migration [15]. In A-PRF, cells are more uniformly distributed inside the clot, also contain an increased count of platelets, and also include more neutrophils. This study compared CAF with A-PRF for Miller's class-II localized gingival recession.

## Materials and methods

Source of data

Patients who attended the OPD of the Department of Periodontology at Bharati Vidyapeeth (deemed to be a university) Dental College, Sangli, were classified according to Miller's classification of localized gingival recession for this study. Ethical clearance was approved by the institute with ref no: BV(DU)MC&H/IEC/2019-20/D-21.

Study design

Twenty patients were included in the research; all of them had Miller's class II localized gingival recession. Participants were recruited from among those who had already arranged an oral assessment with the researcher and were followed for three months. Patients with localized P. D. Miller's type II gingival recession who were willing to give informed written permission and complete the research term were eligible for inclusion. The following conditions disqualified patients from participation in the study: severe allergies to any of the drugs used in the treatment, pregnancy or lactation, and sites that had previously had mucogingival therapy; smokers, in addition to the existence of cervical caries.

Clinical parameters

At the start, after one month, and again after three months, we documented and analyzed all the clinical data. The UNC-15 periodontal probe was used for all the measurements. Recession depth (RD) was measured from the center of the buccal cemento enamel junction to the free gingival margin. The width of keratinized gingiva (KT) is the distance between the mucogingival junction and the free gingival border mid-buccally. Using the rollover method, the keratinized tissue that forms the mucogingival junction was located by rolling the mucosa until it became immovable. To calculate the percentage of root coverage, multiply [RD preoperative - RD postoperative] by 100%. Plaque index (Silness and Loe, 1964): "The oral hygiene status was evaluated by the presence or absence of visible plaque present at the soft tissue margin."

Presurgical therapy

All enrolled individuals also had a standard blood check and radiographs. Initial treatment included education on how to best care for one's teeth and gums, as well as any necessary scaling, root planing, or occlusal modifications were performed. A periodontal re-evaluation was conducted three weeks after the first phase of treatment.

A-PRF preparation

**Figure 1:**
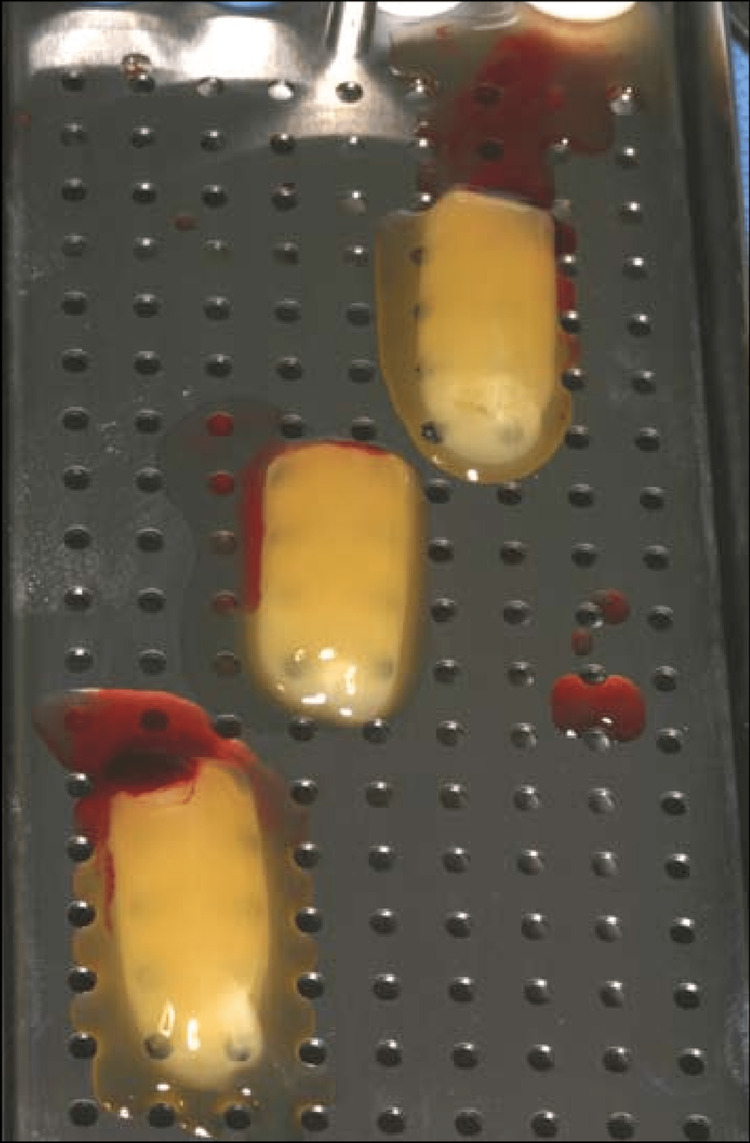
PRF membrane prepared

Venipuncture of the antecubital vein yielded 10 ml of blood, which was collected in a sterile test tube without anticoagulant or other artificial biochemical alteration. Quickly, blood was centrifuged at 1500 rpm for 14 minutes in a centrifuge machine by SHK-Quantos (China). Platelet-poor plasma (PPP) was found at the top of the centrifuged sample, followed by platelet-rich fibrin in the center, and finally by red blood cells at the bottom of the tube. With a pipette, we scraped off the topmost layer of PPP, and was discarded. A pair of pliers was placed into the tube and used to carefully grip the fibrin clot with the blood clot attached to it. The fibrin clot was placed in the PRF box, and the clot was scraped off carefully before being disposed of. The box was then covered with a lid. To ensure a steady flow of serum into the metal container, the box was left undisturbed (Figure 1).

Surgical therapy

**Figure 2:**
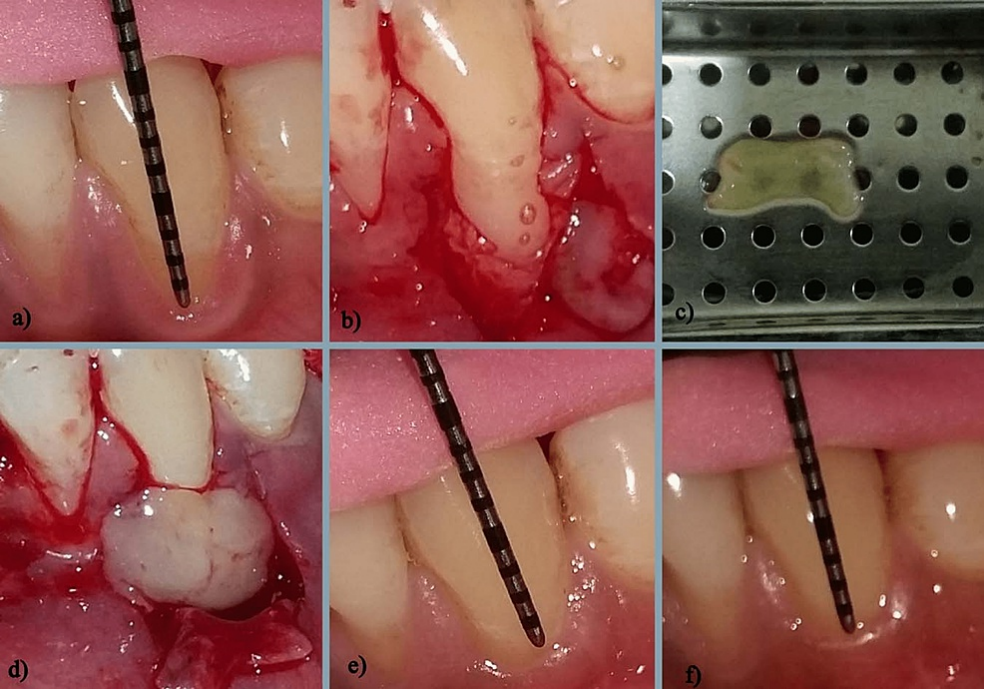
Steps of the surgical procedure

For this study, twenty people with Miller's class II localized gingival recession were chosen by the Periodontics Center at the Bharati Vidyapeeth (deemed to be a university) Dental College, Sangli. Patients were allocated at random to the "test" or "control" group. Group A (the control group) and Group B (the test group) had a coronally advanced flap operation. Before completing an informed consent form, patients were given thorough explanations of the treatment procedure. We recorded every aspect of our subjects' medical and dental histories, down to the presence or absence of any absolute or relative contraindications. After the pre-surgery phase was over, 20 patients were chosen for the study. The selected quadrant was divided into two groups. Control Group (Group A): In the control group, patients were treated with a coronally advanced flap without A-PRF. Test Group (Group B): In the test group, patients were treated with a coronally advanced flap with A-PRF (Figure 2).

Local anesthetic was administered first. The split-thickness flap was then prepared after making an intra-crevicular incision. On either side of the teeth that needed to be treated, an incision was made at the level of the CEJ of the interdental papillae from the distal ends of the horizontal incisions, and vertical releasing incisions were made to the lining mucosa. In a split-full-split thickness pattern, the flap was raised. To make a tissue bed, interdental papillae were de-epithelialized. The PRF was developed to cover the root surface to the CEJ level and extended about 3 mm beyond the osseous defect borders in the CAF + PRF group. The PRF was stabilized using the sling suture method.

Sling sutures were used to coronal advance and fix the flap to the level of the CEJ. The PRF was entirely covered. The vertical incisions were closed with interrupted sutures. Except for where the PRF was placed, the surgical procedures were the same in the CAF group.

Statistical analysis

For the statistical analysis, IBM Corp. Released 2011. IBM SPSS Statistics for Windows, Version 20.0. Armonk, NY: IBM Corp. was used. The statistical techniques used were as follows: When two means from the same group or two values from the same sample were drawn from a normal distribution, the unpaired t-test was employed to compare the means. Statistical significance was determined by the p-value, which had a 95% confidence interval and a p-value of less than 0.05.

## Results

Plaque index (PI)

Unpaired t-tests were used to compare plaque indices in the control and test groups, with a p-value of 0.05 indicating statistical significance, > 0.05 indicating no difference, and 0.001 indicating a highly significant difference. Over three months, the average value in the control group dropped dramatically from 2.0 ± 0.0 to 0.99 ± 0.03 (Table 1).

**Table 1:** Intergroup comparison of mean plaque index score between Group A (Control group) and Group B (Test Group) NS: Not significant, SD: Standard deviation

	Group A (Control group) Mean (SD)	Group B (Test Group) Mean (SD)	Unpaired t-test	Significance
Baseline	2.0 ± 0.0	1.9 ± 0.31	t = 1.00	p =0.331 (NS)
1 month	1.3 ± 0.48	1.0 ± 0.0	t = 1.964	p =0.065 (NS)
3 months	0.99 ± 0.03	1.0 ± 0.0	t = -1.00	p = 0.331 (NS)
Change from Baseline to 1 month	0.7 ± 0.48	0.9 ± 0.31	t = -1.095	p =0.288 (NS)
Change from 1 month to 3 months	0.31 ± 0.49	0.0 ± 0.0	t = 1.961	p =0.066 (NS)
Change from Baseline to 3 months	1.01 ± 0.03	0.9 ± 0.31	t = 1.095	p =0.288 (NS)

At three months, the mean value in the test group dropped from 1.9 ± 0.31 to 1.0 ± 0.0. Values from baseline to three months did not change significantly between the two groups (Table 1).

Recession depth (RD): The decline magnitude between the control and test groups was analyzed using an unpaired t-test, with a p-value of 0.05 indicating statistical significance, > 0.05 showing no difference, and 0.001 indicating a very significant difference. From 2.39 ± 0.81 mm at the gauge, the average RD in the control group decreased to 0.6 ± 0.84 mm after one month and to 0.36 ± 0.61 mm after three months. The test group's initial characteristics were 3.18 ± 1.36 mm, dropping to 0.41 ± 0.51 mm after one month and then to 0.15 ± 0.47 mm after three months. This reveals a statistically significant difference (p 0.05) between the first and third months post-measure in RD (Table 2).

**Table 2:** Intergroup comparison of mean recession depth (in mm) between Group A (Control group) and Group B (Test Group) NS: Not significant, SD: Standard deviation

	Group A (Control group) Mean (SD)	Group B (Test Group) Mean (SD)	Unpaired t- test	Significance
Baseline	2.39 ± 0.81	3.18 ± 1.36	t = -1.570	p =0.134 (NS)
1 month	0.6 ± 0.84	0.41 ± 0.51	t = 0.607	p =0.552 (NS)
3 months	0.36 ± 0.61	0.15 ± 0.47	t = 0.878	p = 0.391 (NS)
Change from Baseline to 1 month	1.79 ± 0.78	2.77 ± 1.15	t = -2.221	p =0.039*
Change from 1 month to 3 months	0.23 ± 0.41	0.26 ± 0.36	t = -0.149	p =0.883 (NS)
Change from Baseline to 3 months	2.02 ± 0.76	3.03 ± 0.99	t = -2.534	p =0.021*

Width of keratinized Gingiva (KT): The decline magnitude between the control and test groups was evaluated using an unpaired t-test, with a p-value of 0.05 indicating statistical significance, > 0.05 showing no difference, and 0.001 indicating a very significant difference. The average KT in the control group had increased from 2.08 ± 0.57 to 2.71 ± 0.59 after one month and then to 3.1 ± 0.65 after three months. The test group had increased from 1.43 ± 0.62 to 2.38 ± 0.56 after one month and then to 3.0 ± 0.00 after three months. But non-significant changes were seen between the control and test group post-measure from baseline to three months follow-up (Table 3). 

**Table 3:** Intergroup comparison of mean width of keratinized gingiva between Group A (Control group) and Group B (Test Group) NS: Not significant, SD: Standard deviation

	Group A (Control group) Mean (SD)	Group B (Test Group) Mean (SD)	Unpaired t- test	Significance
Baseline	2.08 ± 0.57	1.43 ± 0.62	t = 2.429	p =0.026*
1 month	2.71 ± 0.59	2.38 ± 0.56	t = 1.276	p =0.218 (NS)
3 months	3.1 ± 0.65	3.0 ± 0.0	t = 0.361	p = 0.722 (NS)
Change from Baseline to 1 month	0.63 ± 0.4	0.95 ± 0.49	t = -1.585	p =0.130 (NS)
Change from 1 month to 3 months	0.38 ± 0.39	0.61 ± 0.58	t = -1.047	p =0.309 (NS)
Change from Baseline to 3 months	1.01 ± 0.66	1.57 ± 0.83	t = -1.644	p =0.118 (NS)

Percentage of root coverage percentage (%)

The difference in root coverage between the control and test groups was analyzed using an unpaired t-test, with a p-value of 0.05 indicating statistical significance, 0.05 indicating no difference, and 0.01 indicating an extremely large difference. In the month after treatment, 89.16 ± 17.76% of areas had achieved mean root inclusion in the control group. The average score increased from 89.06 ± 15.47 at the 30-day mark to 98.8 ± 3.79 at the 90-day mark. Accordingly, there was not a significant difference between the control and test groups (Table 4).

**Table 4:** Intergroup comparison of mean percentage of root coverage in % between Group A (Control group) and Group B (Test Group) NS: Not significant, SD: Standard deviation

(in percentage)	Group A (Control group) Mean (SD)	Group B (Test Group) Mean (SD)	Unpaired t test	Significance
Baseline	100 ± 0.0	100 ± 0.0	t =0.00	p = 1.000 (NS)
1 month	89.16 ± 17.76	89.06 ± 15.47	t = 0.013	p =0.989 (NS)
3 months	95.9 ± 8.71	98.8 ± 3.79	t = -0.965	p = 0.347 (NS)
Change from Baseline to 1 month	10.83 ± 17.76	10.93 ± 15.47	t = -0.013	p =0.989 (NS)
Change from 1 month to 3 months	-6.73 ± 11.87	-9.73 ± 15.82	t = 0.479	p =0.638 (NS)
Change from Baseline to 3 months	4.1 ± 8.71	1.2 ± 3.79	t = 0.965	p =0.347 (NS)

## Discussion

Due to the growing interest in aesthetic dentistry, gingival recession therapy is gaining prominence in clinical periodontology [16]. The diagnosis of the gingival recession may be made easier with the help of many categories that have been presented in the literature by Sullivan & Atkins [17]; Mlinek et al. [18]; Miller et al. [19]; Smith et al. [20] and Mahajan et al. [21]. Miller's Classification remains the most used system of its kind today. Murphy claims that an assessment was made of Miller's categorization and that it was found to be acceptable. It relies on a morphological assessment of the damaged periodontal tissues and has the potential to foretell how much root coverage will occur once a free gingival graft is performed. Using the findings from the analysis of the soft and hard periodontal tissues, we were able to classify recession abnormalities into four distinct groups.

The most typical method of surgical root covering is the coronally advanced flap [22]. When the remaining gingiva is substantial in thickness and breadth, most investigations support the concept that treatment with CAF alone may be done effectively [23]. Thus, locations with thin residual gingiva may be the only ones eligible for adjunctive use of a graft. Thus, A-PRF was employed in conjunction with CAF in the current investigation. PRF also encourages a quicker bond to the tooth with a more permanent outcome. Blood activation is slowed by PRF, which may increase leukocyte degranulation and cytokine release from pro-inflammatory mediators like interleukin (IL)-1, IL-6, and tumor necrosis factor- to anti-inflammatory cytokines like interleukin (IL)-4, as well as growth factors like transforming growth factor-, platelet-derived growth factor-, and vascular endothelial growth factor, and glycoproteins seem to have a major influence on a variety of healing processes, such as growth factor production, immunological regulation, anti-infectious action, and matrix remodeling. Matrix proteins essential for cell migration are impregnated into the root surface, and growth factors are uniformly dispersed over its surface to stimulate gingival connective tissue (fibronectin, vitronectin, and thrombospondin-1). In addition to its mechanical adhesive properties, the fibrin matrix also exhibits biological activities comparable to those of fibrin glues, such as preventing necrosis and shrinking of the flap, promoting neoangiogenesis, and ensuring total root coverage [8].

Combining full and split flaps has several positive aspects. The thickness of the apical split is increased to aid in the flap's coronal displacement, and the thickness of the coronal full-thickness section, which includes the periosteum, is increased to improve the predictability of root coverage [16]. This study's results demonstrated improved gingival phenotype at test locations, as measured by the thickness of the gingiva. Experiments on monkeys have shown that thin biotype gingiva at locations of alveolar bone dehiscence may act as a factor for the development of soft tissue recessions [24]. Growth factors released by PRF may have stimulated the growth of fibroblasts in the gingiva and periodontal ligament, leading to an increase in GT, or the PRF membrane may have created space between the fibroblasts, leading to an increase in GT [8]. Treatment of several adjacent recession defects is difficult because, to reduce patient suffering, at least two recessions must be handled within a single surgical session. The recommended methods for treating numerous gingival recessions are CAF, modified CAF, and the modified coronally advanced tunnel approach [1]. These procedures produce good cosmetic results because the soft tissue used to cover the exposed root is identical to that which was initially present at the buccal aspect of the tooth with the recession defect. The method used in this study to treat multiple gingival recessions was called CAF. Utilizing the material's alleged benefits, such as promoting fibroblast activity and enhancing angiogenesis on soft tissue healing, platelet-rich fibrin was utilized in conjunction with other substances to treat defects [8,9]. Dixit et al. [25] treatment of gingival recession with either modified CAF or PRF alone or in combination was examined in split-mouth research. When comparing the recession coverage between the control and experimental groups, no significant differences were seen. There seems to be a considerable increase in gingival thickness once PRF is added, which may enhance the predictability and longevity of soft tissue root covering, as mentioned by the author, which is concordance with the present study, where after the CAF, there is improvement in gingival thickness and root coverage, but the result was not significant between the groups. Kurien et al. [26] examined the difference between using CAF alone and using PRF along with it. The mean GR (before treatment) for the test group was 2.66 ± 0.86, whereas the GR (before treatment) for the control group was 2.77 ± 0.87. They found that the CAF+PRF group saw a dramatic rise in GT. A greater percentage of root coverage postoperatively in the experimental group was cited as evidence of the therapeutic benefit of the PRF membrane. Root coverage and gingival width were both positively affected by both treatment methods in the current investigation. While the recession depth improvement was seen significantly between the groups. No statistically significant difference in root coverage was seen between the two groups. The clinical result may have been different if the current research had evaluated other variables, such as PRF consistency or platelet concentration. In addition, the healing process wasn't analyzed histologically. As a result, we don't yet know how much of an impact PRF has on how well connective tissue attaches.

## Conclusions

Miller class II localized gingival recessions were successfully covered by the roots using both the CAF + A-PRF and CAF methods. The additional use of an A-PRF membrane did not provide additional benefits in terms of root coverage outcomes compared with CAF alone. The use of A-PRF membranes significantly reduced the recession depth. For the treatment of numerous gingival recessions, PRF may be an option for several grafting materials.
